# The Era of Nanomaterials: A Safe Solution or a Risk for Marine Environmental Pollution?

**DOI:** 10.3390/biom11030441

**Published:** 2021-03-16

**Authors:** Maria Consiglia Esposito, Ilaria Corsi, Gian Luigi Russo, Carlo Punta, Elisabetta Tosti, Alessandra Gallo

**Affiliations:** 1 Department of Biology and Evolution of Marine Organisms, Stazione Zoologica Anton Dohrn, Villa Comunale, 80121 Napoli, Italy; mariaconsiglia.esposito@szn.it (M.C.E.); glrusso@isa.cnr.it (G.L.R.); elisabetta.tosti@szn.it (E.T.); 2 Department of Physical, Earth and Environmental Sciences, University of Siena, Via Mattioli 4, 53100 Siena, Italy; ilaria.corsi@unisi.it; 3 Institute of Food Sciences, National Research Council, 83100 Avellino, Italy; 4 Department of Chemistry, Materials, and Chemical Engineering “G. Natta”, Politecnico di Milano and INSTM Local Unit, Via Mancinelli 7, 20131 Milano, Italy; carlo.punta@polimi.it

**Keywords:** nanomaterials, nanoremediation, marine pollution, environmental remediation, ecotoxicology, eco-design, ecosafety, ecological risk assessment

## Abstract

In recent years, the application of engineered nanomaterials (ENMs) in environmental remediation gained increasing attention. Due to their large surface area and high reactivity, ENMs offer the potential for the efficient removal of pollutants from environmental matrices with better performances compared to conventional techniques. However, their fate and safety upon environmental application, which can be associated with their release into the environment, are largely unknown. It is essential to develop systems that can predict ENM interactions with biological systems, their overall environmental and human health impact. Until now, Life-Cycle Assessment (LCA) tools have been employed to investigate ENMs potential environmental impact, from raw material production, design and to their final disposal. However, LCA studies focused on the environmental impact of the production phase lacking information on their environmental impact deriving from in situ employment. A recently developed eco-design framework aimed to fill this knowledge gap by using ecotoxicological tools that allow the assessment of potential hazards posed by ENMs to natural ecosystems and wildlife. In the present review, we illustrate the development of the eco-design framework and review the application of ecotoxicology as a valuable strategy to develop ecosafe ENMs for environmental remediation. Furthermore, we critically describe the currently available ENMs for marine environment remediation and discuss their pros and cons in safe environmental applications together with the need to balance benefits and risks promoting an environmentally safe nanoremediation (ecosafe) for the future.

## 1. Introduction

Environmental pollution results from the rising of industrial activities and urbanization, which constantly discharge man-made wastes into the environment altering its equilibrium, integrity, and health. On daily basis, different pollutants are released into soil, atmosphere, lakes, groundwater and rivers, which in turn reach seas and oceans [1]. Consequently, as a final sink of anthropogenic pollutants, marine ecosystems are under threat and need to be restored to healthy conditions. In this scenario, nanotechnology is the science of the 21st Century that can offer the most promising devices to counteract with chemical pollution, including the marine environmental remediation. Combining physical and chemical laws, nanotechnology is able to manipulate matter generating particles on a scale of less than 100 nm, known as engineered nanomaterials (ENMs) or nanoparticles (NPs) [2].

NPs can be synthesized starting from a wide variety of raw materials by applying biological, physical, or chemical methods. Based on the size of raw material, these methods include two general approaches: (i) Top-down approach starts from bulk material to create correspondingly smaller structures using finer tools; and (ii) Bottom-up approach assembles materials from the nanoscopic scale, such as molecules and atoms, to form larger structures [3,4].

Protocols for ENMs/NPs synthesis are improving, promoting their environmental application that refers to “the reduction or removal of contaminants from polluted media restoring their original status” [5,6]. The use of ENMs for environmental remediation, known as nanoremediation, is more effective compared to conventional remediation approaches since nanometric materials show high reactivity, high surface-area-to-volume ratio, and a target-specific ability to capture toxic compounds [7]. Additionally, the latter approach allows a faster degradation and stabilization of contaminants by ENMs reducing the time frame and even the costs of the process [8,9,10]. Moreover, ENMs are more sustainable because they minimize the addition of chemicals, reduce the amount of material needed in the clean-up process [11], and potentially extend the range of available in situ remediation technologies [12,13]. Nano-sized particles are transferred into contaminated media such as soils, sediments, and aquifers by in situ remediation technology. This strategy is preferred over other approaches being more cost-effective. In most applications, zero-valent iron nanoparticles (nZVI) are successfully used to remediate groundwater, soil, wetland and river sediments [14,15,16,17]. Furthermore, the effectiveness of other ENMs for in situ remediation of soil, groundwater and sediments has been demonstrated [18,19,20].Therefore, application methods for in situ treatment with ENMs may also be suitable for the deployment in the marine environment.

To date, experimental studies established that ENMs can be employed to efficiently restore different marine environmental matrices [21,22,23], reducing the impact of toxic chemicals, thus, preserving marine biodiversity, ecosystem functioning and services. However, as such, they are applied as primary ENMs to the environment and the balance between benefits and risks associated with their use is still under debate [24,25]. Their potential release in the environment and the deriving effects on the ecosystem health become a matter of concern to be addressed. For this purpose, it is necessary to elucidate the fate and behavior of ENMs, which depend not only on their physical and chemical proprieties, but also on the characteristics of the receiving environment [26]. Of particular interest is the marine environment, which appears as a dynamic and extreme environment, due to the high ionic strength conditions, pH, and presence of a high amount of naturally occurring particulates. Indeed, upon entry into the marine environment, ENMs undergo diverse processes, such as dissolution, transformation, speciation, agglomerate/aggregate and sedimentation, which may influence their fate and dispersion and determine the bioavailability and toxicity. These research areas need further investigation to establish a proper environmental risk assessment following an in-depth understanding of the ENMs physico-chemical properties.

Moreover, in order to achieve a “green and sustainable remediation” (GSR), it is essential to assess the risks posed by the ENMs applied to marine environment remediation techniques [27]. As a future goal of the remediation industry, environmental safety represents the main challenge for ENMs employed in marine nanoremediation and can be achieved by using environmental risk assessment approaches [24]. In such a way, the ecotoxicological testing strategy represents a fundamental aspect since it adapts the standardized ecotoxicity tests, or newly developed ones, allowing the determination of the potential impact of ENM/Ps toward different levels of biological organization, thus providing suitable toxicity data. This information will help to identify the ENMs properties that mediate the interaction with living organisms and, consequently, their toxicity, leading to the selection of the best ecofriendly and ecological sustainable ENMs [28].

In the present work, we will review the development of the eco-design framework and the application of ecotoxicology as a valuable strategy to develop ecosafe ENMs for environmental remediation. Furthermore, we critically describe the currently available ENMs for marine environment remediation and discuss their pros and cons in safe environmental applications together with the need to balance benefits and risks promoting a future environmentally safe nanoremediation (*ecosafe*).

## 2. ENM/NPs and Environmental Safety

ENMs have been identified as innovative tools to deal with the global concern of marine pollution. The best performances and higher sustainability of the ENMs, compared to macro-sized materials, are driving the progressive transition from remediation to nanoremediation. Several studies demonstrated the efficacy of ENMs in the decontamination of the marine polluted sites. However, more efforts are needed to unravel any potential environmental implication deriving from their use due to the documented toxicological outcomes on marine biota [24,28,29]. Therefore, it is crucial to develop an ecosafety strategy to protect the marine biota before the authorization of applications for in situ nanoremediation of the marine environment. A key issue in designing ENMs regards their fate after release. This aspect acquires more relevance since, once released in seawater, ENMs undergo significant transformations, which affect their behavior and toxicity on marine biota. According to the Scientific Committee on Emerging and Newly Identified Health Risks [30], chemical-physical properties of ENMs (size, surface composition, shape, solubility, aggregation, chemical reactivity) are fundamental in the risk assessment. In fact, the intrinsic characteristics of ENMs, together with the properties of the environmental matrices, are among the factors that can induce transformations of ENMs, and, consequently, affect their potential risks for human and ecosystems health [31,32,33]. Marine environment is an alkaline medium characterized by a high ionic strength and a wide variety of natural organic matter (NOM) [26]. ENMs can undergo dissolution into an ionic form driven by the particle chemistry, but they also rapidly co-aggregate (homoaggregation) or assemble with non-homologous particles (heteroaggregation) due to the high ionic strength and the relatively high pH of sea water. The ENM aggregation increases their size; consequently, the aggregates are less mobile and tend to be deposited to the sediments, becoming less available to organisms in the water column [34]. ENMs in marine water can also interact with the inorganic and organic colloidal particles, resulting in a greater stabilization effect on NPs that can influence their aggregation dynamics and colloidal stability.

The interaction between ENMs and NOM in the aquatic matrices is emerging as an attractive research field. Recently, it has been suggested that NOM produced during the algal bloom may contribute stabilize ENMs, limiting the agglomeration process [35]. However, depending on the media proprieties, the bio-nano interaction can induce an opposite mechanism, promoting agglomeration [36,37].

**Figure 1:**
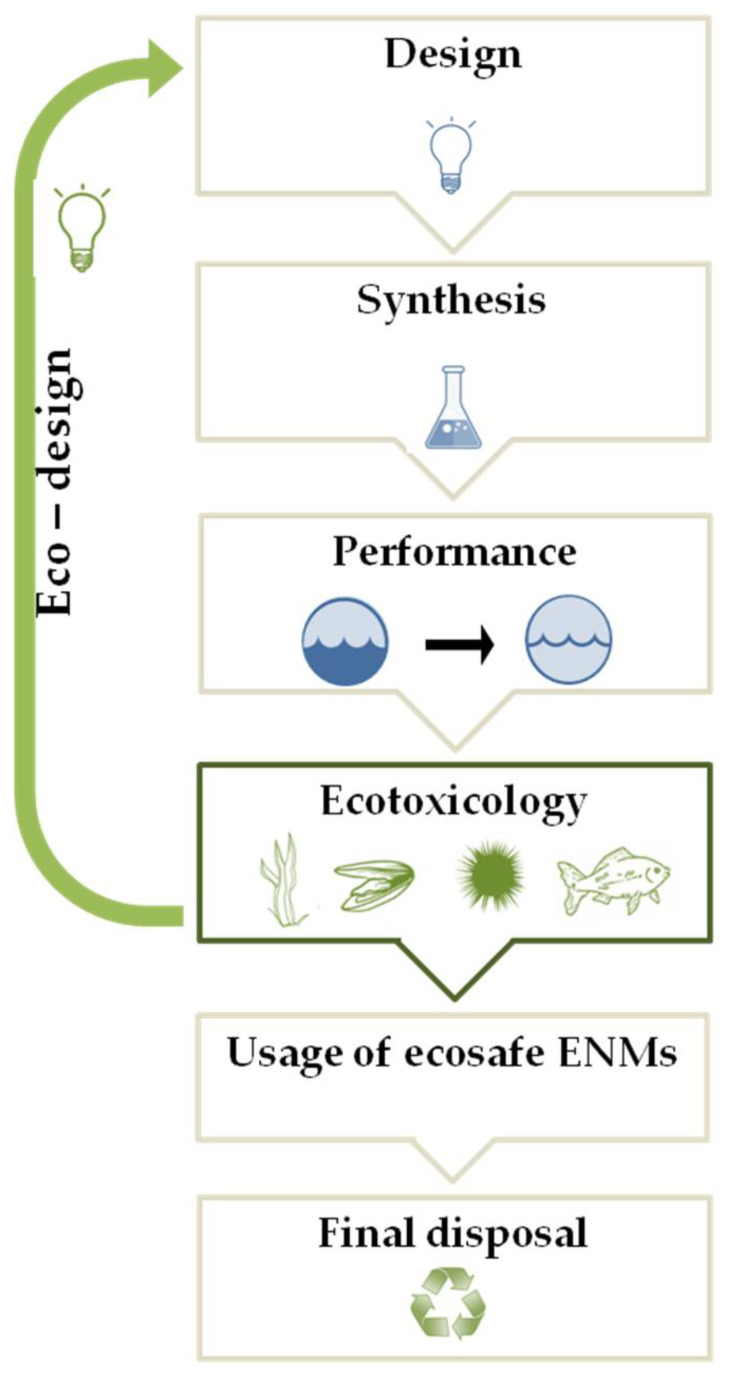
A schematic representation of the eco-design approach (see text for details).

In some condition, the interaction of NPs with dissolved biomolecules can favor the formation of NOM-related nanoscale coatings, analogous to protein corona in mammalian systems, potentially affecting aggregation and transport of the NPs, as well as bio-distribution, uptake and toxicity to marine species [38,39,40,41]. Alternatively, NPs can be adsorbed on the exterior surface of the organism driving surface inducing toxicity. The aggregation and adsorption processes can increase the ENM concentrations in the environmental matrices. Overall, once released in the marine environment, NPs may undergo rapid transformations due to their intrinsic and extrinsic properties, which drive NP fate and determine their ecotoxicity on the marine biota [38,42]. Despite the sign of progress in the research studies of the environmental fate and behavior and risk assessment of ENMs, to date, their life cycle is characterized by a regulatory gaps, from the design and synthesis, to their usage, until the final disposal. Specific international regulation for producing, labeling, and evaluating the environmental impact of ENMs is lacking [43]. Attempting to fill these gaps, in 2007, the European Commission introduced the ENMs in the register of chemical compounds REACH (Registration, Evaluation, Authorization and Restriction of Chemicals) indicating that their safety assessment should follow the risk assessment methodology adopted for conventional chemicals. Based on the purpose of promoting green nanotechnology, which does not pose any risk for the environment and biota, progresses in nanoremediation are moving towards the design of eco-friendly nanosized devices with a low content of toxic substances, reduced material and energy requirements. Following this perspective, the United States Environmental Protection Agency published multiple documents as reference points, including the best management practice fact sheets for green and sustainable remediation [44]. Aiming to reduce the environmental impact of the ENMs and promote sustainable frontiers for nanoremediation, the “eco-design” approach (Figure 1) is gaining relevance.

This innovative approach is aimed to keep under control the environmental safety of new ENMs through the “safety-by-design” strategy (Figure 1) that results safe and sustainable in terms of ENM composition, production process, and performance. Therefore, ecotoxicology plays a key role in the “eco-design” approach in order to determine the potential risk of new synthesized ENMs for in situ nanoremediation in the marine environment [24,45]. The ecotoxicological assessment of ENMs can change the design and/or the composition of an ENM, realizing the eco-design of newly synthesized ENMs aimed at safeguarding the environment during the complete ENMs life-cycle, until their final disposal, when the nanoremediation process is over, producing recyclable and/or biodegradable ENMs, supporting the development of green nanotechnologies for marine remediation.

### Ecotoxicological Assessment of ENMs

From the first evidence of ENM impact on aquatic species, nanoecotoxicology gained a relevant role in ecological risk assessment (ERA) of ENMs by recognizing how their transformations in sea water (i.e., size distribution, surface charges and bio-nano interactions) affected biological interactions and toxicological responses at population and ecosystem level [28,29,31,46]. Nanoscale dimension represents the main driver of cellular uptake, but exposure scenarios are affected by ENM transformations occurring in natural environments, which also identify potential target ecosystems (pelagic versus benthic) [47]. Linking exposure to the observed biological effects is a key aspect for proper ERA and bio-nano interactions are fundamental for the understanding of such complex natural exposure scenarios [28]. Eco-corona formation, as a results of particle physical-chemical interaction with dissolved biomolecules already existing in natural seawater, will affect particle uptake and related cellular pathways leading to toxicity [24,29,31,47]. To make regulatory references more suitable, a general agreement has been reached by the nanoecotox scientific community in using more realistic exposure scenarios for ERA of ENMs and in revising current standardized protocols based on bioassays [28,33,48,49]. Although effect-based tools including in vitro and in vivo bioassays have been successfully used to assess exposure and hazard for legacy and emerging marine pollutants, they present some limitations for ENMs [49,50,51]. Conversely, conventional biomarkers, such as those developed upon exposure to other toxicants (e.g., oxidative stress, lipid peroxidation, biotransformation and genotoxicity) have been successful to assess ENM effects at cellular level and identify common biological pathways, or determine toxicity [52,53]. Although not specifically modulated by ENM exposure, these biomarkers have been extremely helpful to identify cellular pathways affected by ions dissolution from NPs (i.e., AgNPs) or ENM interaction with biomolecules (i.e., DNA damage and neurotoxicity). More recently, autophagy, lysosomal dysfunction, and immunomodulation, all conserved mechanisms from invertebrates to mammals, are emerging as biomarkers for the early interactions between cells and ENMs [52,53,54,55]. Furthermore, the modern field of ecotoxicogenomics is promising in understanding the mode of action of ENMs and even in the recognition of adverse outcome pathways [56]. This will allow the identification of the first warning response upon ENM exposure and predict consequences on higher biological organization (i.e., from cell up to organs, organism and population) and marine taxa for an overall ecosystem assessment [57]. To this aim, a multi-biomarkers approach and integrate individual biomarker response indices has been proposed in order to limit any risk associated with over/underestimation of the observed biological effects [2,52,58,59,60,61].

Although standard test guidelines developed for conventional contaminants have been used to test ENMs, concerns have been raised on their appropriateness for addressing particle properties under different testing conditions and assessing the toxic effects. Due to the peculiar characteristics of ENMs, several issues must be taken into consideration including the behavior of ENMs in exposure media.

## 3. ENMs Employed for Marine Environment Remediation and Their Ecosafety

Marine environmental remediation can be achieved by different conventional methods and technologies, such as coagulation, precipitation, filtration, in situ burning of the oil spill, sediment-capping, and mechanical removal (ex situ treatments). Both the production process and the application of the traditional methods employed to clean polluted marine area need a huge amount of time, money, energy, and give rise to wastes that often cannot be regenerated. These issues can be overcome by the application of nano-based techniques, which offer more effective alternatives to traditional methods of seawater treatment. The ENM production processes can be simpler, limit the wasteful secondary reactions, and can reduce energy consumption with benefits for the environment and workers’ health [62].

Furthermore, ENMs can remove contaminants at lower concentrations compared to the traditional methods [63]. The higher performance of nanoremediation depends on the peculiar physical-chemical properties of ENMs. These can be grafted with functional groups increasing the sensitivity, the target selectivity, the timing, and the efficiency of the nanoremediation process [64,65]. The higher selectivity along with the few compounds employed during the production process causes a reduction of the wastes produced after remediation treatment and boosts the reuse or the recycling of the contaminant specifically removed [66,67]. However, despite the social, economic, and environmental benefits of nanoremediation, its application is scarce due to the lack of a comprehensive assessment of the environmental risks related to ENMs.

In fact, compared to the number of different ENMs synthesized for seawater decontamination (Table 1), studies assessing the toxicity of each single ENM are scarce (Table 2). Similarly, limited interdisciplinary investigations are present in the current literature on the remediation ability of new synthetized ENMs and the ecotoxicological impact on marine organisms. This stimulate a more efficient interaction between different research fields, such as chemistry, physics, engineering, and ecotoxicology, and improved research efforts on the ecotoxicological assessment of ENMs. To obtain safe ENMs, ecotoxicological tests should mimic the real conditions before and after the nanoremediation process, taking into consideration the peculiar characteristics of ENMs, such as different sizes, structures, and shapes that contribute to the interactions with the remediation media, affecting their behavior and toxicity.

**Figure 2:**
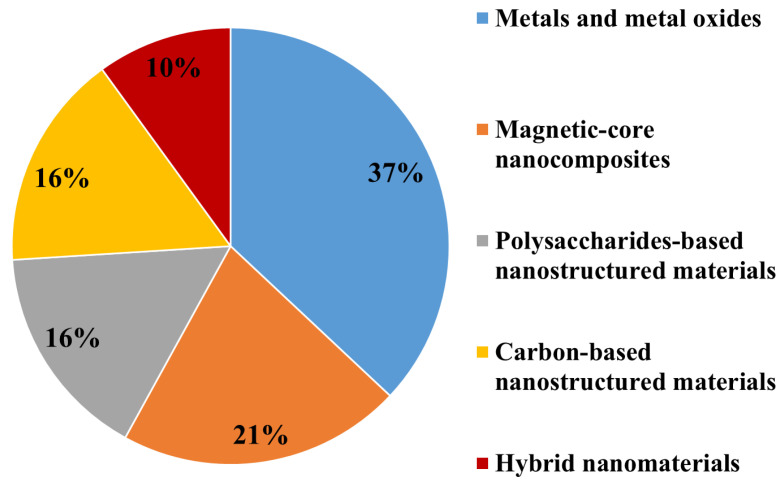
Classes of ENMs employed for marine environment nanoremediation.

Based on their main chemical compositions, ENMs used for marine environment nanoremediation can be broadly grouped as: (i) Metal oxides based nanomaterials (the most abundant class, 37%); (ii) magnetic-core nanocomposites (21%); (iii) carbon-based and polysaccharides-based nanostructured materials employed at the same percentage (16%); (iv) hybrid nanomaterials, the less employed for marine clean-up (10%) (Figure 2).

**Figure 3:**
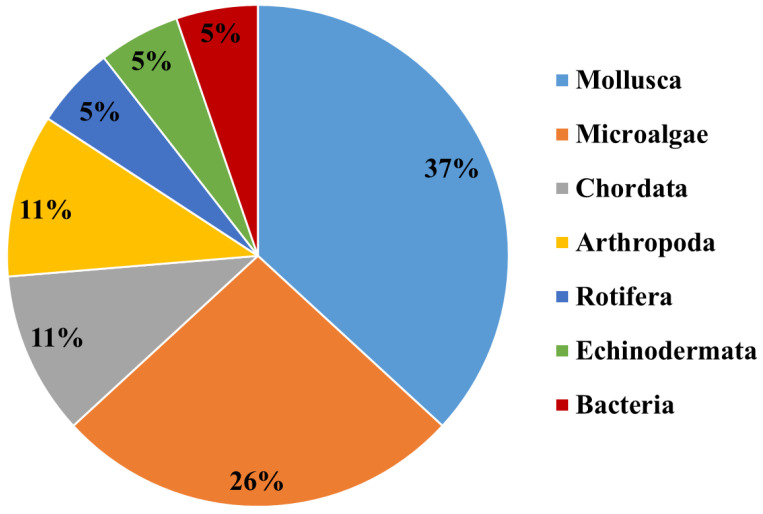
Marine organisms employed for ecotoxicological assessment of ENMs in marine remediation applications belong to the Kingdoms and Phyla indicated in the pie chart (see text for details).

In the following sections, we describe the characteristics of these classes of ENMs designed for marine nanoremediation and their ecotoxicological assessment, when available, using the marine organisms belonging to the classification reported in Figure 3, and the exposure conditions mimicking the marine environment.

### 3.1. Metal and Metal Oxides Based Nanomaterials

Metals are widely employed as base materials to produce ENMs. In fact, metal-based ENMs are characterized by a good capacity in removing heavy metals and organic pollutants from water due to their fast kinetics and high adsorption efficiency [94].

Adsorption is a mechanism broadly used both in traditional remediation approaches and nanoremediation for treating contaminated seawater. For instance, since 2013, the adsorbent capacity of functionalized ferrite NPs has been investigated for Pb and Hg removal from seawater [72,95]. Nowadays, the latest advancement in the application of ferrite oxides for marine nanoremediation is focused on manganese-ferrite NP as a good adsorbent material [96]. Recently, Coppola and co-workers explored the use of manganese-ferrite NP (MnFe_2_O_4_-NPs, produced by the oxidative hydrolysis of ferrous sulfate heptahydrate and manganese sulfate in alkaline conditions) to remediate seawater from As and Pb [72,73]. Despite their efficiency for metal remediation in seawater, these ENMs have been demonstrated to negatively affect marine bivalve species (*Mytilus galloprovincialis*, *Ruditapes philippinarum*) causing a reduction of the metabolic activity and a gain of antioxidant defenses, neurotoxicity, and oxidative stress [71,72,73]. Further studies are necessary on marine organisms of different trophic levels to assess the safety of MnFe_2_O_4_-NPs for in situ marine remediation application.

The zero valent nanomaterials (nZVs) have been considered as promising ENMs for environmental remediation being high reactivate in adsorbing and degrading different contaminants. nZVs have been synthesized from various naturally reducing agents and are able to transform organic (trichloroethane, trinitrotoluene, pesticides, dyes), or inorganic contaminants (heavy metals and inorganic anions) into less harmful or harmless substances [97]. Nanoscale zero-valent iron (nZVI) is one of the main candidates for environmental nanoremediation, being able to replace organic and inorganic pollutants in the environment using iron widely distributed in nature [98,99]. Compared to iron particles, nZVI possesses several advantage, such as a fast kinetics, high reactivity, high removal capacity, and the possibility to be injected directly into a contaminated site due to its proprieties, such as small particle size, high surface-area-to-volume ratio, magnetism [100]. The latter represents a useful property for water treatment systems since it does not provide attractive magnetic forces between nanoparticles and allows separation and recovery of magnetic nanoparticles from solution to be reused applying methods that are cost effective, i.e., a magnetic field or a hand-held magnet [101]. Nevertheless, further researches are needed to better understand the potential application and the environmental risk of nZVI. In fact, to date, only a few studies assessed the toxicity of nZVI in marine organisms. In particular, nZVI has been demonstrated to cause a decrease in the growth rates in three species of phytoplankton [74] and a delay in embryo development in three free spawning invertebrates (*M. galloprovincialis*, *Ciona intestinalis*, and *Psammechinus milliaris*) [91,92].

Nano-iron oxide (nFe_3_O_4_) and nFe_3_O_4_/fly ash composites have been investigated to remediate contaminated seawater from triphenyltin chloride (TPT), an organotin compound employed as pesticides and fungicides. Their adsorption efficiency was examined at different pH, temperature, and mass of adsorbent nanocomposite employed and tested on seawater collected from a contaminated harbor. The functionalized Fe_3_O_4_ with fly ash nanocomposite revealed a higher ability to remove TPT compared to nFe_3_O_4_ [75]. However, the ecotoxicological assessment of such ENMs has not been yet performed.

Titanium dioxide NP (nTiO_2_) has gained considerable attention in marine environmental remediation as an efficient catalyst and adsorbent of organic contaminants and heavy metals. The toxicity of nTiO_2_ in marine species has been extensively tested in invertebrates and fishes, as well as in marine mammals. In the mollusk abalone (*Haliotis diversicolor supertexta*), nTiO_2_ affected embryo development causing hatching inhibition and malformations [102]. In the mussel *M. galloprovincialis*, nTiO_2_ exposures induced Ti accumulation in a tissue-specific manner, histomorphological and histochemical alterations in gills and digestive gland, and DNA damage in hemocytes [103]. Furthermore, it causes a chromosomal alteration in peripheral erythrocytes in the European sea bass *Dicentrarchus labrax* [104] and DNA damage in bottlenose dolphin (*Tursiops truncatus*) leukocytes [105]. On the contrary, nTiO_2_ particles with a size of 25 nm have been reported to be non-toxic for the bacteria *Vibrio fischeri*, crustaceans and rotifers (*Artemia salina* and *Brachionus plicatilis*), suggesting a minimal risk for marine organisms when used for remediation purpose [106].

Only recently, ecosafety of silver NPs (AgNPs) bifunctionalized with hydrophilic capping agents as citrate (Cit) and L-cysteine (L-cys), developed as sensor of mercury but also able to remove it from polluted waters, have been demonstrated [107]. Exposure of two microalgae, the marine *Phaeodactylum tricornutum* and the freshwater *Raphidocelis subcapitata* to increasing concentrations (10–500 µg/L) of AgNPs coated with Cit and L-cys showed no effects on algal growth, supporting the protective role of capping agents in preventing the release of Ag ions from the NP in both freshwater and seawater media. This study confirmed the absence of risks associated with AgNPs future environmental applications as sensor for Hg and for its removal [107]. AgNPs ecotoxicity has been largely documented both in terrestrial and aquatic environments and on various taxa from bacteria to fish including mammalian cell lines [38,108,109,110,111,112]. The release of Ag^+^ from dissolved NPs in exposure media has been recognized as a driver to explain the observed toxicity; however, a nanoparticle-based effect has been also reported [50,51,52,54,58,113,114]. Therefore, an ecosafety assessment of AgNPs for environmental applications is mandatory and can be achieved using bioassays as suitable tools to support their use *in situ*.

Similarly, nanomaterials based on hexacyanoferrate (HCF), a dark blue pigment also named Prussian Blue (PB), have been examined for the remediation of cesium (Cs) polluted seawater [115,116,117]. In particular, copper hexacyanoferrate (CuHCF) NPs containing K^+^ ions (KCuHCF-NPs) show chemical stability in artificial seawater and high efficiency and sensitivity; furthermore, they can be easily recovered from seawater by filtration, after the coagulation-precipitation method [76]. The last advancement in seawater remediation from radioactive Cs is represented by the zeolitic imidazolate framework (ZIF-8) functionalized by ferrocyanide (FC). The adsorption ability of ZIF-8-FC has been tested at various pH and temperatures and an excellent Cs^+^ selectivity has been demonstrated in artificial seawater (ASW) [77]. However, to date, the absence of toxicity on marine organisms represents a limitation for a safe environmental application.

### 3.2. Magnetic-Core Nanocomposites

Magnetic particles are particularly attractive due to their superparamagnetic nature and unique physical-chemical properties, such as high dispersibility, relatively large surface area, and the high ratio of surface to volume resulting in a higher adsorption capacity. They are characterized by a shell with a core, which consists of magnetic elements such as iron, nickel, cobalt, or their oxides [118]. Among them, polyvinylpyrrolidone (PVP)-coated iron oxide NMs have demonstrated to be able to remediate seawater from different metals and oils [82]. Furthermore, the oil removal efficiency of these magnetic NMs can be increased combining their activity with oil-degrading bacteria [83]. This finding underlines the possibility of a merged application that includes nanoremediation and microbial bioremediation for in situ treatments. The acute toxicity of PVP-Fe_3_O_4_NMs has been tested in the copepod *Amphiascus tenuiremis* demonstrating that the optimal concentration for oil removal did not affect copepod survival [93]. However, the toxicity of the optimal concentration for metal removal and the chronic toxicity have not been yet evaluated. PVP-coatedFe_3_O_4_ NMs can be produced by a simple and cost-effective method of hydrothermal synthesis, which requires no organic solvents, low toxic reactants, and low temperature/energy [93,119]. This feature, along with the demonstrated effectiveness at the lab-scale and the absence of acute toxicity, encourages future experiments at a larger scale and an ecotoxicological assessment that includes different levels of biological organization and chronic exposure.

The removal of radioactive Cs^+^ from seawater was also investigated in a study where researchers synthesized core-shell multilayer magnetic microspheres [78]. The Fe_3_O_4_@SiO_2_@KTiFC magnetic microparticles demonstrated removal efficiency in different natural seawater conditions and high selectivity for Cs^+^ and other ions, such as Na^+^, Ni^2+^, Fe^3+^, Sr^2+^, Mo^6+^, Zr^4+^, Ba^2+^, and Nd^4+^ supporting their application in the decontamination of Cs^+^ radioactive seawater, even if, to date, their ecotoxicity remains unexplored.

The latest progress in the treatment of Cs-contaminated seawater underline the role of the Prussian blue-embedded magnetic hydrogel beads (PBMHBs) as recoverable adsorbent [23]. PB presents a high selectivity for radioactive Cs, which confers to these beads a high efficiency in removing it from seawater. Among the benefits of PBMHBs, it can be mentioned the eco-friendly and simple one-step protocol developed to encapsulate PB in MHBs and the low cost for possible large-scale treatment of Cs-contaminated water. Moreover, the ability of nanosized PB to act as ROS scavengers has been demonstrated by in vitro studies that support the possible ecosafety of this nanomaterial [120]. However, to our knowledge, no studies have been published on its impact on marine biota.

Magnetic carbon microspheres (CMs) coated with nFe_3_O_4_ have been used to efficiently degrade polycyclic aromatic hydrocarbons (PAH) from contaminated marine sediments, from which they can be easily recycled since CMs exhibit an excellent response to a magnetic field [79]. For these ENMs, to date, the potential impact on marine biota is unknown.

### 3.3. Hybrid Nanocomposites

Carbon-based ENMs are giving a significant contribution to the development of functional and suitable materials for marine remediation. They are characterized by a scaffold of carbon atoms that can assume different three-dimensional structures generating fullerene, carbon nanotubes (CNTs), graphene, and graphite. Many decades have passed since, in 1985, the chemists Kroto and colleagues casually discovered a spherical structure of 60 carbon atoms [121]. Fullerene is characterized by peculiar properties, such as chemical stability, high electron affinity and high surface to volume ratio, which make it a useful nanomaterial to be applied in the production of nanofiltration (NF) membranes [122]. Indeed, compared to the microporous membrane, NF carbon-based membranes are characterized by smaller pores, which enhance their permeability to water and gas making them a promising tool for seawater clean-up [123]. Moreover, some NF membranes exhibit an antifouling ability, which promotes their longer life-span, reducing the energy consumption of the remediation process. Whereas, the apolar and hydrophobic character of fullerene can facilitate ENM recovery after remediation. Currently, most NF membranes are thin-film composites (TFC), and their ability to selectively separate ion metals (Li^+^, Mg^2+^, Pb^2+^, Cd^2+^, Zn^2+^, and Ni^2+^) from aqueous media has been widely investigated [124,125]; however, to date, it has been synthesized only one membrane that exhibited a high Mg^2+^/Li^+^ separation factor suggesting its potential application in treatment of Li contaminated seawater [22]. CNTs are one-dimensional fibrous nanomaterials formed by a single or multiple rolled layer, which assumes the structure of a hollow and cylindrical tube [126]. They can be easily functionalized for a specific filtration or sorption of inorganic contaminants from seawater and their adsorption efficiency is pH-dependent [127]. CNTs have been also developed to treat spilled oil. Oil run-off from offshore platforms or accidental tanker spills results in the formation of a sticky crude oil layer on the sea surface [128]. The addition of dispersants to facilitate the dispersion of the floating oil layer into smaller droplets is a traditional method for mitigating oil spill impacts, but the majority of dispersants are toxic to humans and aquatic species. Trying to obtain a green oil spill clean-up, different complementary studies are moving toward the synthesis of dispersant-free ENMs or ENMs with a lower content of dispersants. Surfactant-loaded halloysite nanotubes (HNTs) show the potentiality to replace traditional liquid dispersant formulations enhancing marine oil spill remediation [129,130,131]. Other cheap and environmentally sustainable alternatives to face oil spill clean-up are the three-dimensional (3D) aerogels and carbon-based sponges that can be obtained through different procedures [132,133,134]. Compared with traditional sorbents, the resulting 3D materials exhibit a larger oil sorption capacity, high hydrophobicity and oleophilicity, mechanical stability, large surface areas and are reusable and/or recyclable [135,136,137,138,139]. Recently, modified extended graphene (M-EG) with cetyl trimethyl ammonium bromide (CTAB) and potassium bromide (KBr) has been synthesized and demonstrated to be efficient in removing different oils from seawater at different sodium salt concentrations. However, its adsorption ability was affected by temperature [140]; finally, its capacity to be regenerated several times by filtration-drying cycles, without altering its adsorption performance, makes it an ideal candidate for treating marine oil pollution in practical application. Jiang et al. [68] designed a graphene oxide (GO) sponge enriched with florin groups which enhance the sponge hydrophobicity preventing the entry of water into the pores. The hierarchically porous sorbent material is able to adsorb organic solvents and various oils and to retain its adsorption efficiency for a long time, reducing time and materials consumption of the oil recovery process. An additional material employed for marine environmental remediation is the graphene oxide chitosan-based (GO-CH-based). It is a hydrogel with a 3D macrostructure developed taking advantage of the ability of GO to self-assembly in presence of the natural biopolymer such as chitosan [141,142]. Chitosan is a non-toxic and hydrophilic compound deriving from the alkaline deacetylation of chitin, which boosts the adsorption ability of the hydrogel. Nevertheless, GO-CH nanocomposites was not efficient in removing Hg from seawater [141]. On the other hand, chitosan-grafted carbon nanotubes (CTS-g-CNTs) are gaining more attention as a viable material to remedy contaminated seawater from radioactive Cs. The higher presence of –OH functional groups, related to the grafted chitosan, increases the interaction with Cs^+^ ions and the removal efficiency, which is pH-dependent and relies on the presence of the competitive cations [69]. The literature herein reviewed provides the high efficiency of this class of ENMs in seawater remediation; but, on the other hand, it does not face the issue of ecotoxicological assessment, posing a limit for their application in real scenarios.

An exception is represented by the GO functionalized with polyethyleneimine (GO-PEI), which is able to decontaminate seawater from Hg. Indeed, the toxic impact of this nanostructured material has been evaluated in mussel (*M. galloprovincialis*) resulting in necrosis and apoptosis in mature oocytes and histopathological damages in the digestive tubules [70].

### 3.4. Polysaccharides-Based Nanostructured Materials

Carbohydrates (e.g., starch, cellulose, glycogen) are gaining the attention of researchers to produce innovative and ecosafe nanostructured materials. The preparation of polymer nanocomposites using nanosized starch and cellulose is of growing interest in environmental remediation due to the unique characteristics of these nanomaterials, which possess the potential to overcome challenges of toxicity, biodegradability, renewability, accessibility, cost, and energy consumption [42,143,144,145,146,147]. Starch is a mixture of two polymers, the linear amylose and the branched amylopectin. Nanoparticle deriving from native starch can be obtained through chemical, physical, and enzymatic routes [148]. For instance, the enzymatic degradation of starch operated by bacteria leads to the formation of cyclodextrins composed of a hydrophobic cavity and an external hydrophilic surface. Owing to their three-dimensional ring structure, cyclodextrins can encapsulate other molecules making them useful to be applied in the adsorption of toxic pollutants [149,150].

Among the several types and sizes of starch derivatives, nanostructured sponges have been proposed to be among the most promising adsorbents showing good potential for the removal of heavy metals from seawater [84]. Two starch derivatives, the β-cyclodextrin (β-CD) and ^®^linecaps (^®^LC), have been employed as polymer backbone to synthesize different starch-based nanosponges (NSs). NSs were able to capture metals at different test concentrations, both in ultrapure water and seawater, showing a different adsorption ability, which was higher for the citrate NSs compared to the pyromellitic NSs. Additionally, NSs can be easily recovered from treated water by filtration. This first study on the efficiency of starch-based NMs in seawater provides the basis for future researches on the assessment of their toxicity on marine organisms [84].

Besides starch, cellulose is one of the most abundant biopolymer sources on the Earth. It is a high molecular weight crystalline homopolymer composed of a linear chain of β (1→4) linked anhydrous D-glucose units, which can be extracted from a wide variety of sources, including marine animals, plants, bacteria and algae [151]. It can be also obtained from biomass derived from agricultural or food waste, lowering the impact on the use of this raw material, according to the rules of the circular economy. The cleaving of the hierarchical structure of cellulose, where single chains have meshed into fibers, leads to single cellulose nanofibers [152], characterized by at least one dimension under 100 nm [153].

**Figure 4:**
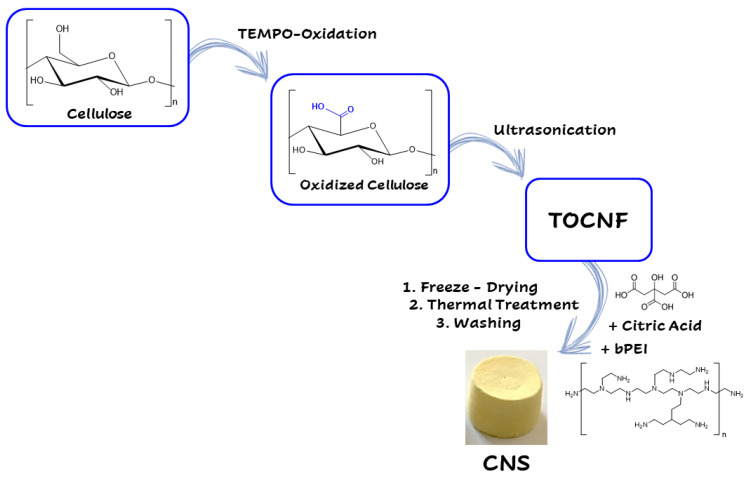
Synthetic steps in the synthesis of CNS (see text for description)**.**

Nanocellulose can be obtained via mechanical processing, hydrolysis (enzymatic or acid), and oxidation mediated by 2,2,6,6 tetramethylpiperidinyloxyl (TEMPO) [154,155]. In 2015, a new class of ENMs, the Cellulose-Based Nanosponges (CNS), was developed by using a two-step protocol (Figure 4).

**Figure 5:**
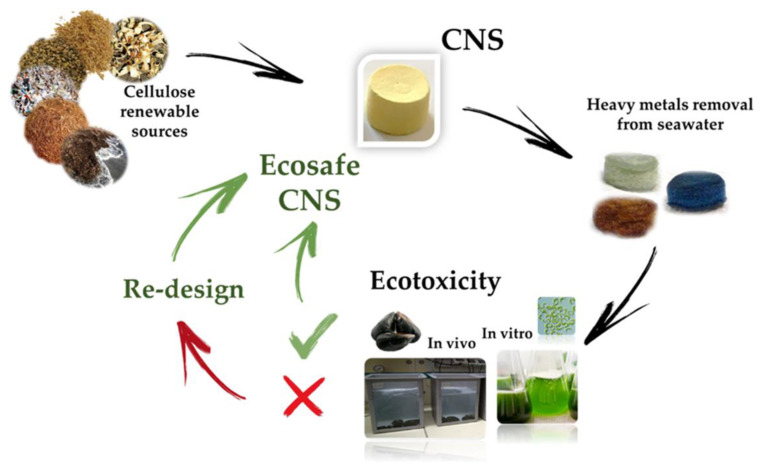
Eco-design of nanostructured cellulose sponges (CNS) for the remediation of heavy metal polluted seawater (modified by [45]).

The first step foresees the production of cellulose nanofibers (CNF) by following the TEMPO-mediated oxidation protocol [156]. Subsequently, the alcoholic groups in the C6 position of glupyranosic rings are partially converted to the corresponding carboxylic groups. Ultrasonication or homogenization at basic pH favors the defibrillation of cellulose fibers to provide the TEMPO-oxidized CNF (TOCNF), taking advantage from the electrostatic repulsion among deprotonated (and negatively charged) carboxylic moieties. The second step consists of the addition of branched polyethyleneimine (bPEI) and citric acid (CA) to a 2% *w/w* water suspension of TOCNF. The resulting highly viscous hydrogel is transferred into well-plates, used as molds, and undergoes thermal treatment consisting of: (i) freezing, (ii) lyophilization, and (iii) heating in oven at about 100 °C. This procedure aims to promote the cross-linking of nanofibers by the formation of amidic bonds between the carboxylic groups present TOCNF backbone and the amine moieties of the bPEI polymer. The resulting CNS evidences a nanoporous structure characterized by a high chemical and mechanical stability [86]. CNS, used as it is or ground into powder, have demonstrated high efficiency to remove heavy metals (Zn, Cd, Cr, Hg, Ni, Cu) and organic dyes from water and artificial seawater [45,85,89,157,158]. More importantly, these nanostructured materials have been synthesized and optimized following the lab scale, the eco-design concept previously discussed, in order to ensure the eco-safety of the nanosponge, while preserving its remediation efficiency (Figure 5).

The ecotoxicological evaluation of CNS class has been performed using species representing two levels of the marine trophic chain: the alga *Dunaliella tertiolecta* and the mussel *M. galloprovincialis*, representing the primary producer and the primary consumer, respectively, in the trophic chain. CNS did not affect the growth rate of the marine alga, supporting the ecosafety of these ENMs and promoting its application in nanoremediation [45]. However, the bPEI, a component of CNS, was able to reduce the algal growth. Hence, different formulations of CNS have been re-designed by changing the amount of two key components of CNS, bPEI and citric acid. Among the new formulations, the CNS with the following components mass ratio: bPEI/TOCNF/CA 1:1:18% ensured both the remediation efficiency in ASW and the ecosafety in algae and mussels [45]. In a different study, the cellular and tissue responses in mussels were measured after 48 h exposure in four different experimental conditions: ASW, Zn contaminated ASW, CNS, and ASW after CNS treatment [89]. The cellular bioassays showed that the lysosomal membrane stability, the frequency of nuclear abnormalities, the DNA integrity and apoptotic cells in gill, the frequency of micronucleus in hemocytes were not affected in mussels exposed to CNS. Moreover, the genotoxic, cytotoxic, and histological damages induced by Zn were fully recovered following CNS treatment. Altogether, these results support the CNS ability to remove Zn from ASW and their ecosafety [89].

Carbohydrates are commonly used also for gel preparations. Recently, a nanostructured-cellulose hydrogel was able to remove efficiently radioactive Cs from seawater maintaining its adsorption stability for a long time [87]. The super adsorption efficiency of nanocellulose hydrogels in the removal of dyes or oil-spilled from water and the potentiality of polysaccharides-based nanocomposites for environmental remediation have been demonstrated [159,160,161,162]. However, additional efforts are required to implement the knowledge on their performances and ecotoxicological impact.

### 3.5. Composite-Based Nanomaterials

Ongoing innovation for an ecosafe nanotechnology generated new solutions deriving from the integration of carbon-based, metal-based, or polysaccharides-based NMs. Consequently, the border which separates the classes is labile. Therefore, the classification of NMs described above should not be considered conclusive. Combining the benefits of magnetic PB and GO, Yang and coworkers [88] designed an exceptional nanocomposite material for the removal of radioactive Cs from water, the PB/Fe_3_O_4_/GO nanocomposites, characterized by a low aggregation rate, a high adsorption surface area and high stability. Furthermore, considering the main components of these innovative ENMs, the PB/Fe_3_O_4_/GO nanocomposites possess all the characteristics to be a cheap, reusable, and eco-friendly material for Cs-decontamination in the marine environment, but the ecotoxicological assessment remains to be explored.

## 4. Conclusions

Nanotechnology has grown exponentially in the last decades, resulting in the production of huge amount of ENMs for multiple applications. The improved ability to manipulate matter has driven the progressive interconnection of nanotechnology with environmental remediation, until the recent application of ENMs for marine environmental remediation. ENMs applications provide new possibilities to face environmental challenges since they have demonstrated high effectivity in the degradation and/or removal of contaminants. Nanoremediation techniques are more effective in time and costs, respect to conventional remediation methods [10], but the main challenge is to develop ENMs able to remove contaminants from environmental matrices (soil, air, and water) utilizing natural and renewable sources, to safeguard environment and consequently human health. Nanotechnology offers safe and green approaches, which can revolutionize the environmental remediation techniques by preventing the formation of secondary by-products and decomposing some of toxic pollutants with zero waste. However, there are several uncertainties regarding the fundamental features of this technology, which have made it difficult to engineer applications for optimal performance or to assess the risk to human or ecological health. In the future, since it is expected that several nanotools will be developed and applied for environmental remediation, it will be mandatory to closely monitor their ecosafety.

The environmental hazard assessment of each new synthetized ENM, as well as the supervision of the whole life cycle of ENMs for marine nanoremediation, shows some weaknesses, which lead to the following considerations. The use of ENMs for salt-water clean-up made little and slow progress compared to their application in other fields, or in water treatments. Most of the studies on ENMs for marine environmental remediation does not progress until the pilot-scale or over, but it is stacked at the lab-scale. One reason that gets slower the practical application of nanoremediation in the marine environment is the absence of a shared project with manufacturing industries. Hence, better-coordinated communication between research entities, governments, and industries should be implemented with the objective to boost clean energy and sustainable technologies.

In relation to the ecotoxicological evaluation of ENMs for marine nanoremediation purposes, in situ investigation of ENMs, long-term and chronic studies should be incremented over short-term and acute ecotoxicity tests. To our knowledge, studies in marine matrix lack data on some trophic levels being mainly focused on primary producers and consumers, such as algae and mussels. Additionally, the majority of ecotoxicological investigations are concentrated on biomarkers in adults, while little attention has been given to reproductive efficiency, which determines the survival or extinction of a given population. Compared to non-aquatic organisms, the external fertilization that is present in some key marine species directly exposes the gametes and the early developmental stages to contaminants. Hence, from an ecological perspective, we would like to propose the reproduction as a biomarker of exposure and effect to ENMs in marine environment [163]. On the other hand, ecotoxicogenomic approach reflects the latest efforts to find sensitive biomarkers to ENM exposure, contributing to gain a complete understanding of the action of ENMs to marine organisms.

The last advances in the biomarkers and tools, together with the new knowledge in the environmental behavior of ENMs, could integrate future nanoecotoxicology studies for an efficient ecological risk assessment, able to describe more realistically the ENM exposure scenarios and to predict ecosystem impact caused by ENM application.

Finally, we would like to point out the need for a standardized approach to nanosafety that integrates the chemical, physical and biological aspects. To date, the eco-design strategy seems to face this challenge for green and safe nanomaterials.

## Figures and Tables

**Table 1:** ENMs synthetized for the remediation of marine environment.

ENM	Concentration	Properties	Target Contaminants	Mechanism	Media	Remediation Efficiency	Reference
Graphene oxide sponge enriched with florin groups (USTC-6@GO@sponge)	NF	carbon-based ENM with microporosity and great hydrophobicity for the selective adsorption of organic compounds	diesel oil, gasoline, soybean oil, light petroleum, n-hexane, bromobenzene, N’N-dimethylformamide (DMF), tetrahydrofuran, acetone, CCl_4_ methylbenzene	adsorption	seawater	NF	[68]
Chitosan-grafted carbon nanotubes (CTS-g-CNTs)	0.6 g L^−1^	external nanotube diameter of 30 nm and an inner diameter of 8.48 nm, stable in seawater	Cs	adsorption	seawater	NF	[69]
Graphene oxide functionalized with polyethyleneimine (GO-PEI)	10 mg L^−1^	foam with three dimensional porous structures	Hg	adsorption	seawater	NF	[70]
Manganese-ferrite NPs (MnFe_2_O_4_)	50 mg L^−1^	NP diameter of 75 ± 15 nm; magnetism	As, Pb	adsorption	seawater	NF	[71,72,73]
Alginate and polyvinyl alcohol (PVA)-alginate entrapped nanoscale zero-valent iron (nZVI)	1 g L^−1^ 2 g L^−1^	Particles of powder average size 50 nm	Cu, Zn, Cr, As	adsorption	saline wastewater	Cu 84.2%; Cr 70.8%; Zn 31.2%; As 39.8%	[74]
nFe_3_O_4_/fly ash composite	0.5 g in 25 mL of triphenyltinchloride (TPT) solution	nFe_3_O_4_ size particles < 50 nm	TPT	adsorption	seawater	98.40%	[75]
Potasium copper hexacyanoferrate (KCuHCF)	0.1 g L^−1^	NPs size of 10–17 nm	Cs	adsorption	seawater	99%	[76]
Zeolitic imidazolate framework-8 functionalized with ferrocyanide (ZIF-8-FC)	V/m = 1000 mL g^−1^	cubic particles with a surface area of 589 m^2^ g^−1^	Cs	adsorption	seawater	60% at 3 h 85% at 24 h	[77]
Magnetic multilayer core–shell (Fe_3_O_4_@SiO_2_@KTiFC)	5 mg of Fe_3_O_4_@SiO_2_ @KTiFC particles added to 4 mL seawater	microspheres with a magnetite core of 300 nm; magnetism	Cs	adsorption	seawater	97.7%	[78]
Prussian blue-embedded magnetic hydrogel beads (PB-MHBs)	1 mg mL^−1^	average size of 33.8 mm; magnetism	Cs	adsorption	seawater	96.7%	[23]
Magnetic carbon microspheres (Fe_3_O_4_-CM)	5 g L^−1^	diameter microspheres of ~30 μm; superparamagnetis	polycyclic aromatic hydrocarbons (PAH)	degradation	marine sediments	87%	[79]
Nano-hydroxyapatite particles (nHAp)	0–10% nHAp/dry weight	rod structure with dimensions of 20 nm (i.d.) × 200 nm (length); surface area of 130 m^2^ g^−1^	Pb, Cd	sorption	marine sediments	NF	[80]
nZVI coated to polyacrylic acid (nanofer 25S)	low (2, 3 and 4%) and high (5, 10 and 20%) dosages	diameter of 50 nm; total iron content of 80–90 wt. %; surface area of 20–25 m^2^ g^−1^	Al, As, B, Ba, Co, Cu, Ni	adsorption, reduction	marine sediments slightly polluted by heavy metals	at 3 g: Co 100%; at 4 g: Al 33.3%, As 76%, Cu 96.8%, B 0%; at 5 g: Al 71.4%, Cu 100%, As 62%; At 10 g: B 60.4%; at 20 g: Co 54.3%	[81]
Nanoscale zero valent iron (nZVI)	0.01–1 g/L	particle sizes < 100 nm	polycyclic aromatic hydrocarbons (PAHs)	oxidation	PAHs contaminated sediments	70.2% at 0.01 g/L, 78.3% at 0.1 g/L, 86.3% at 0.5 g/L, 78.0% at 1 g/L	[55]
polyvinylpyrrolidone-coated magnetic ENM (PVP-Fe_3_O_4_ NMs)	167 mg/L	median size of 11.2 nm	Pb, Cr, Ni, Cd	adsorption	seawater	Pb 100%; Cr 98.8%; Ni 60–70%; Cd 40–50%	[82]
	375 ± 10 mg/L		oil-water mixtures			70% of lower-chain alkanes (C9–C22); 65% of higher-chain (C23–C26),	[83]
Starch-based nanosponges	12 mg in 15 mL	citrate nanosponges with β-cyclodextrin (β-CD) or ^®^linecaps (^®^LC) scaffold	Cu, Zn	adsorption	seawater	Cu 80–84% Zn < 60%	[84]
		pyromellitic nanosponges with β-cyclodextrin (β-CD) or ^®^linecaps (^®^LC) scaffold				Cu 36–45%; Zn <60%	
Powder of Cellulose-Based Nanostructured Sponges (CNS)	0.8 mg mL^−1^	particle size range 50 to 400 μm	Zn, Cu, Cr, Cd	adsorption	seawater	90%	[45,85,86]
KCuHCF-cellulose hydrogel	10 mg in 20 mL	Cubic-shaped particles of 10–12 nm	Cs	adsorption	seawater	>90%	[87]
PB coating Fe_3_O_4_ NPs anchored to the surface of the GO sheets (PB/Fe_3_O_4_/GO)	0.05 g of NPs in 30 mL	average size of 17 nm;magnetism	Cs	adsorption	seawater	52.19%	[88]

NF: data not found.

**Table 2:** Ecotoxicological assessment of ENMs synthetized for the remediation of marine environment.

ENM	Concentration	Properties	Experimental Conditions	Species	Effects	Reference
manganese-ferrite NPs (MnFe_2_O_4_ NPs)	50 mg/L	NPs size of 75 ± 15 nm	24 h exposure in ASW (T 17.0 ± 1.0 °C; pH 8.0 ± 0.1; salinity 30 ± 1; photoperiod light/dark 12 h:12 h; continuous aeration)	*Mytilus galloprovincialis*	enhancement of antioxidant and biotransformation enzymes activities; lipids and protein damages; neurotoxicity	[73]
manganese-ferrite NPs (MnFe_2_O_4_ NPs)	50 mg/L	NPs size 75 ± 15 nm	28 days’ exposure in ASW (T 17.0 ± 1.0 °C; pH 8.0 ± 0.1; salinity 30 ± 1; photoperiod light/dark 12 h:12 h; continuous aeration)	*Mytilus galloprovincialis*	depression of metabolic activity, oxidative stress, cellular membrane damage, neurotoxicity	[72]
GO-PEI	10 mg/L	foam with three dimensional porous structures	28 days’ exposure in ASW (T 17.0 ± 1.0 °C; pH 8.0 ± 0.1; salinity 30 ± 1; photoperiod light/dark 12 h:12 h; continuous aeration)	*Mytilus galloprovincialis*	depression of metabolic activity, oxidative stress, cellular membrane damage, neurotoxicity necrosis and apoptosis in female gonads, cellular atrophy in digestive tubules	[70]
GO-PEI	10 mg/L	foam with three dimensional porous structures	28 days exposure in ASW (T 17.0 ± 1.0 °C; pH 8.0 ± 0.1; salinity 30 ± 1; continuous aeration)	*Ruditapes philippinarum*	depression of metabolic activity, oxidative stress, cellular membrane damage; alteration in gills and in digestive tubules	[71]
CNS	1.25 g/L	powder of cellulose-based nanostructured sponges with particle size range of 50 to 400 μm	48 h of exposure in ASW (T 18 ± 1 °C; pH 8 ± 0.1; salinity 40 ± 1)	*Mytilus galloprovincialis*	none in immune and gill cells and mantle	[89]
	1.25 g/L serial diluitions (1:20, 1:10, 1:5, 1:2, undiluited)		72 h of exposure in ASW initial density of 10^4^ cells m/L	*Dunaliella tertiolecta*	algal growth inhibition with undiluited CNS	[45]
Nanofer25S	0.01–100 mg/L	commercial nanoscale zero-valent iron NPs with a size of 80–120 nm	96 h of exposure in NSW (pH 8.1, 20 °C, salinity 34; light:dark cycle 14:10)initial density of 1–2 × 10^5^ cells mL^−1^	*Isochrysis galbana* *Dunaliella tertiolecta* *Thalassiosira pseudonana*	algal growth inhibition at: 3.1 mg/L for *I. galbana;* 1.3 mg/L for *D. tertiolecta*; 0.4 mg/L for *T.* *pseudonana*	[90]
	1.8–10 mg/L	commercial nanoscale zero-valent iron NPs size 80–120 nm	2 h gamete exposure (T 0.5 °C, pH 8.1; salinity 35.1 ± 0.52, for sea urchins and mussels; T 17 ± 0.1 °C, salinity 36 ± 0.02, for sea squirts) egg: sperm ratio 1:1 × 10^6^	Spermatozoa of *Mytilus galloprovincialis, Ciona intestinalis* *and Psammechinus milliaris*	fertilization success decrease; embryo development delay	[91]
nano-Fe_2_O_3_	0; 100; 1000; 10,000 μg/L	size of 50 nm	NSW (T 15 ± 0.5 °C; pH 8.1; salinity 35.1 ± 0.52)	*Mytilus galloprovincialis*	None on embryo development	[92]
PVP-Fe_3_O_4_ NMs	0–100 mg/L	median size of 11.2 nm	96 h exposure in ASW (T 25 ± 1 °C; salinity 30)	*Amphiascus tenuiremis*	None on copepod mortality up to 25 mg/L	[93]

NF: data not found; natural seawater (NSW); artificial seawater (ASW).

## Data Availability

Not applicable.

## Abbreviations

^®^LC	^®^Linecaps
3D	Three-Dimensional
AgNPs	Silver Nanoparticles
ASW	Artificial Sea Water
bPEI	Branched Polyethyleneimine
CA	Citric Acid
CMs	Carbon Microspheres
CNF	Cellulose Nanofiber
CNS	Cellulose-Based Nanosponge
CNTs	Carbon Nanotubes
CTAB	Cetyl Trimethyl Ammonium Bromide
CTS-g-CNTs	Chitosan-Grafted Carbon Nanotubes
CuHCF	Copper Hexacyanoferrate
DOM	Dissolved Organic Matter
ENMs	Engineered Nanomaterials
ERA	Ecological Risk Assessment
GO	Graphene Oxide
GO-CH-based	Graphene Oxide Chitosan-Based
GO-PEI	Graphene Oxide Functionalized With Polyethyleneimine
GSR	Green And Sustainable Remediation
HCF	Hexacyanoferrate
HNTs	Halloysite Nanotubes
KBr	Potassium Bromide
KTiFC	Potassium Titanium Ferrocyanide
LCA	Life-Cycle Assessment
M-EG	Modified Extended Graphene
MnFe_2_O_4_-NPs	Manganese-Ferrite Nanoparticles
NF	Nanofiltration
NMs	Nanomaterials
NOM	Natural Organic Matter
NPs	Nanoparticles
NSs	Nanosponges
nTiO_2_	Titanium Dioxide Nanoparticles
nZVI	Nanoscale Zero-Valent Iron
PAH	Polycyclic Aromatic Hydrocarbons
PB	Prussian Blue
PBMHBs	Prussian Blue-Embedded Magnetic Hydrogel Beads
POPs	Persistent Organic Pollutants
REACH	Registration, Evaluation, Authorization And Restriction Of Chemic
TEMPO	2,2,6,6 Tetramethylpiperidinyloxyl
TFC	Thin-Film Composites
TOCNF	TEMPO-Oxidized CNF
TPT	Triphenyltin Chloride
ZIF-8-FC	Zeolitic Imidazolate Framework (ZIF-8) Functionalized By Ferrocyanide (FC)
β-CD	Β-Cyclodextrin
